# Nanoparticle-based Plasmonic Transduction for Modulation of Electrically Excitable Cells

**DOI:** 10.1038/s41598-017-08141-4

**Published:** 2017-08-10

**Authors:** Parveen Bazard, Robert D. Frisina, Joseph P. Walton, Venkat R. Bhethanabotla

**Affiliations:** 1 Department of Chemical and Biomedical Engineering, College of Engineering, University of South Florida, Tampa, FL-33620 USA; 2 Department of Communication Sciences and Disorders, College of Behavioral & Community Sciences, University of South Florida, Tampa, FL-33620 USA; 3 Global Center of Hearing and Speech Research, University of South Florida, Tampa, FL-33612 USA

## Abstract

There is a compelling need for the development of new sensory and neural prosthetic devices which are capable of more precise point stimulation. Current prosthetic devices suffer from the limitation of low spatial resolution due to the non-specific stimulation characteristics of electrical stimulation, i.e., the spread of electric fields generated. We present a visible light stimulation method for modulating the firing patterns of electrically-excitable cells using surface plasmon resonance phenomena. In *in-vitro* studies using gold (Au) nanoparticle-coated nanoelectrodes, we show that this method (substrate coated with nanoparticles) has the potential for incorporating this new technology into neural stimulation prosthetics, such as cochlear implants for the deaf, with very high spatial resolution. Au nanoparticles (NPs) were coated on micropipettes using aminosilane linkers; and these micropipettes were used for stimulating and inhibiting the action potential firing patterns of SH-SY5Y human neuroblastoma cells and neonatal cardiomyocytes. Our findings pave the way for development of biomedical implants and neural testing devices using nanoelectrodes capable of temporally and spatially precise excitation and inhibition of electrically-excitable cellular activity.

## Introduction

The vast majority of prosthetic devices that are being used today to measure, study, diagnose or restore normal function of partial or completely lost neural or cardiac activity and operate on the principle of electrical stimulation, e.g., cochlear implants for the deaf^1^, with nearly 400,000 deaf people worldwide currently having cochlear implants, retinal implants for the blind^2^, cardiac pacemakers^3^, with about 3 million people worldwide with pacemakers implanted. The electric fields produced by the applied electric currents tend to spread significantly, leading to non-specific stimulation and low spatial resolution. For example, cochlear implants employ an array of tiny electrodes that stimulate different populations of auditory nerve fibers (ANFs) via current pulses. A sound processor analyzes incoming sound, similar to a Fourier analysis, and determines what electrodes are activated. Despite recent technological advancements, current spread limits the effectiveness to optimally stimulate discrete ANFs. So, the processing of sounds with a high frequency content like speech in the presence of background noise, or music, still remains a very important problem to address^4–6^.

Electrical stimulation is used not only for sensory implants, but also, for techniques like electromyography (EMG), a neurological test used to detect and diagnose peripheral neuropathy and related sensorimotor problems, with the annual cost of EMG being approximately 2.8 billion dollars in the US alone^7^. Along with activation and testing, electrical stimulation is used to treat some neurological disorders, where neural inhibition is needed – as employed for treatment of neurological diseases like brain trauma, and for some studies of brain function^8^. Because of such widespread use of artificial neural stimulation, there is a crucial need to look for alternative stimulation methods that would address the issue of specific point stimulation, and be utilized for the development of advanced sensory and neural prosthetic devices.

Nanomaterial-assisted neural stimulation approaches have drawn attention in recent years^9–11^. In these studies, various power sources are employed to activate different localized fields – magnetic, electric, thermal fields around the different nanomaterials, responsible for modulation of cell signals, for example, magnetic fields^12^, ultrasound waves^13^, and laser light (mostly, near infrared and infrared)^14–19^. In light-based nanoparticle stimulation, the localized surface plasmon resonance (LSPR) fields are generated due to strong surface interactions between light and conduction band electrons of metal nanoparticles, leading to potential alternatives to electrical excitation, used in current biomedical implants. To utilize the LSPR fields for cell stimulation, sufficient amount of nanomaterial has to be extremely close to the targeted tissue; various methods have been employed to achieve this like surface modification of nanoparticles, bio-conjugation and local delivery via injection. For instance, Carvalho-de-Souza *et al*.^14^ conjugated Au nanoparticles with three different ligands – Ts1 neurotoxin and two antibodies (targeting TRPV1 and P2 X_3_ channel receptors) respectively, and successfully bound the particles to dorsal ganglion neurons (DRGs), then stimulated the DRGs with 532 nm green laser light. Li *et al*.^16^ utilized photosensitive hydrogel embedded with polypyrrole (PPy) nanoparticles to release biomolecule transmitters (glutamate for excitation & DNQX for inhibition). A 980 nm infrared (IR) laser light was used to excite hippocampal neurons *in-vitro* when glutamate was released and to inhibit responses from the rat visual cortex *in-vivo* when DNQX was released. Yoo *et al*.^19^ coated Au nanorods with polyethylene glycol (PEG) that facilitates binding to the cell membrane and used a 785 nm near infrared laser to invoke inhibition in rat hippocampal tissues. Eom *et al*.^15^ conjugated Au nanorods to rat sciatic nerve and recorded compound nerve action potential, in response to a 980 nm IR laser. Au nanorods were injected into the sciatic nerve using a glass capillary and nerve bundle was excised subsequently. Similarly, Yong *et al*.^18^ incubated primary auditory neurons with silica-coated Au nanorods overnight and excited the neurons with a 780 nm near IR laser. The above mentioned studies have one common feature in their methodologies, i.e., the modification of nano-neural interfaces to achieve the stimulation. The major drawback with alteration of nano-neural surfaces is that *in vivo* translation raises issues regarding unwanted toxicity, repeatability and bio-compatibility. For example, excessive heating by infrared lasers can damage healthy tissues. Hence, there is need to find more viable ways, which minimize collateral damage, to use *in vivo* for translation into new neural prosthetic and testing devices.

Here, we report an Au nanoeletrode (Au nanoparticle-coated glass micropipette) which does not need any surface modification or bio-conjugation for neural stimulation via visible-light lasers. The nanoelectrodes were characterized via electron microscopy and validated for generation of plasmonic responses via light-induced photocurrents and fluorescence quenching experiments as proof of concept before the cellular physiology experiments. Subsequently, we stimulated two different cells, SH-SY5Y human neuroblastoma a cell line that has characteristics of neurons, and neonatal cardiomyocytes, with a nanoelectrode and a 532 nm green laser. These experiments served as initial, *in vitro* proof of concept that wireless nanoelectrodes in combination with visible light can be used instead of electrical electrodes or infra-red (IR) lasers, for precise temporal modulation of neural and cardiac cellular responses. Based on these initial breakthrough results, we visualize that future biomedical implants based on LSPR phenomena using nanoelectrodes and light will give superior spatial resolution and more clinically useful focal stimulation. Implantable electrodes such as cochlear implant electrode arrays, which use polymeric materials are easily designed using the fundamental results demonstrated in this contribution.

## Results

### Nanoelectrode Characterization and Testing

**Figure 1:**
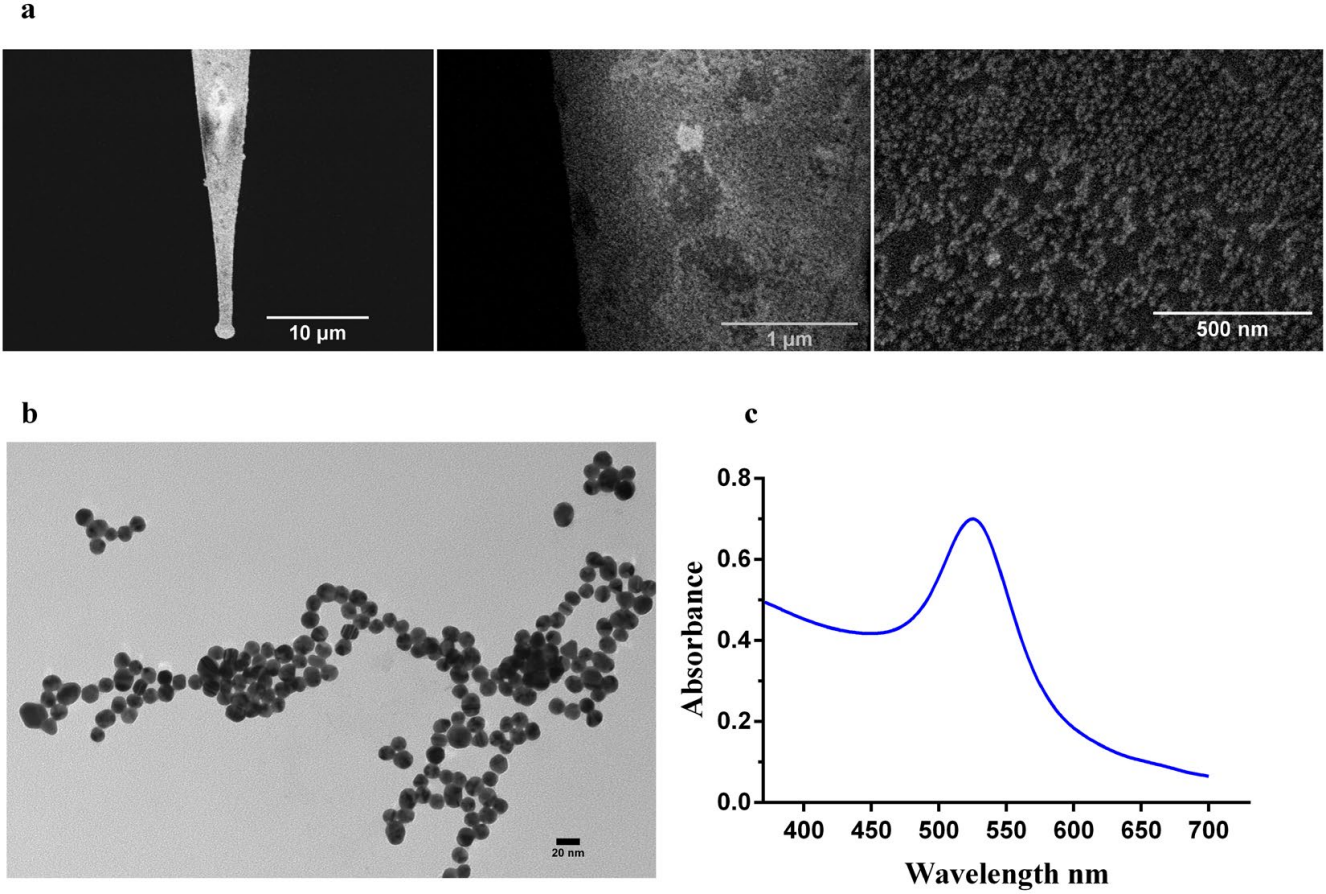
Fabrication of the nanoelectrodes. (**a**) SEM images of an Au nanoelectrode at three magnifications: scale bars 10 μm, 1 μm and 500 nm. The nanoelectrodes used for cell stimulation were fabricated by coating Au nanoparticles onto glass micropipettes, which was facilitated by functionalization of the glass surface with a 10% volume solution of γ –(aminopropyl) tri-ethoxysilane. (**b**) A TEM image of the Au nanoparticles which were synthesized using a liquid phase method. A 2–3 ml solution of 1% trisodium citrate was added to a boiling solution of HAuCl_4_.3H_2_0. (**c**) The LSPR absorption spectrum of the Au nanoparticles.

**Figure 2:**
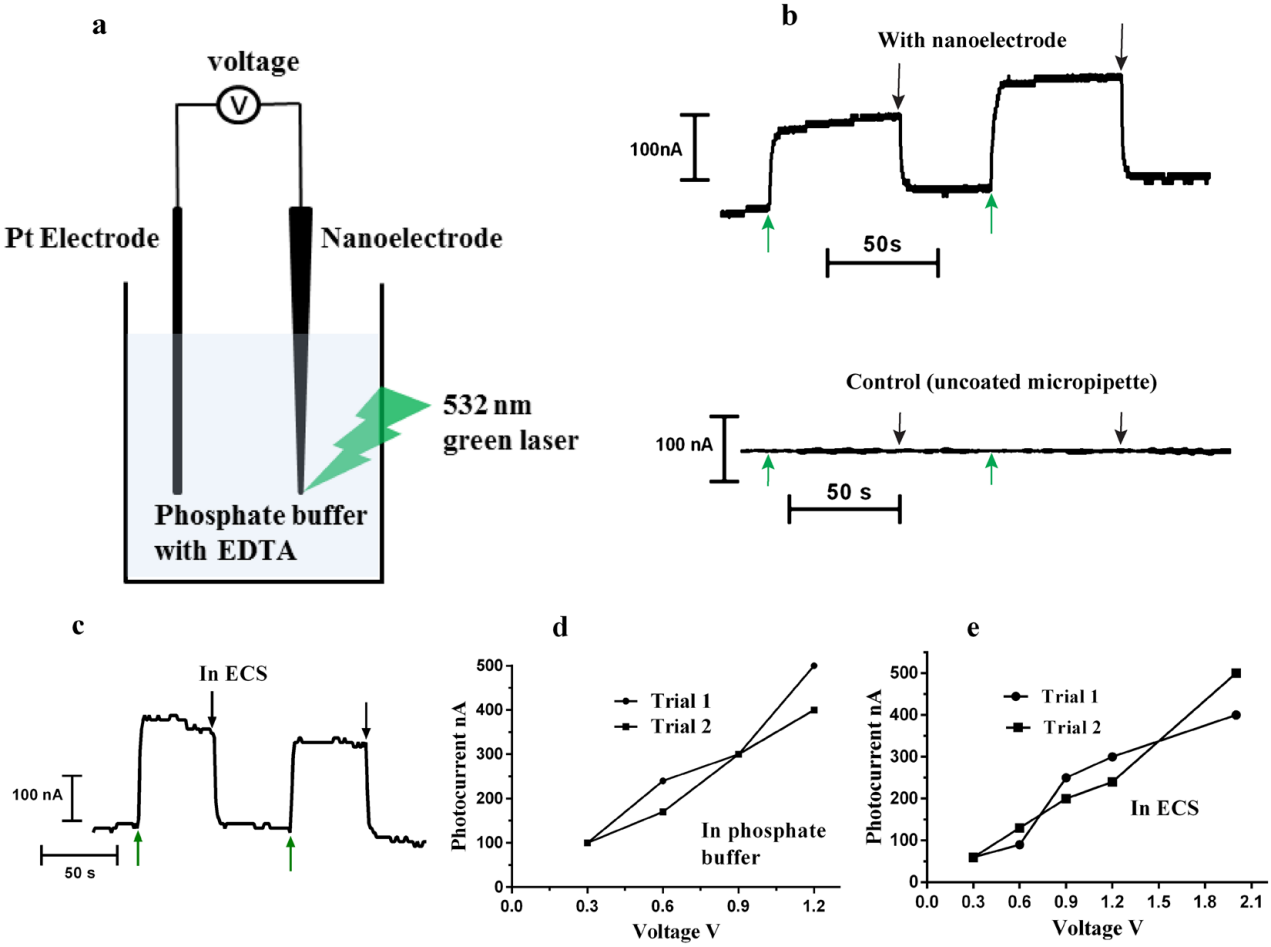
Nanoelectrode testing – Photocurrent measurements. (**a**) Light induced photocurrents were recorded when a nanoeletrode was illuminated with a 532 nm green laser in an electrochemical cell. The experimental set-up schematic is shown in (**a**). A 0.1 M phosphate buffer/0.05 M EDTA solution was used as the electrolyte and standard calomel electrode was used as the reference electrode. (**b**) A 0.3 V vs Ag_(s)_/AgCl, reference electrode was applied. A jump in circuit current was observed when a 532 nm green laser was focused on the tip of the nanoelectrode, shown by the green arrow, and the current dropped back to base values when the laser was switched off, shown by black arrow. (**c)** Photocurrents versus time recordings in extracellular solution when 0.9 V voltage vs Ag_(s)_/AgCl, reference electrode was applied. (**d**,**e**) Photocurrents increase with applied voltage for both electrolytes: 0.1 M phosphate buffer/0.05 M EDTA and NaCl (125 mM) based extracellular solution.

Scanning electron microscopy (SEM) images of the nanoelectrodes (Fig. 1a) show the uniform coating of Au NPs on the outer surface of the electrode tip. Au NPs synthesized via a liquid phase route were characterized using transmission electron microscopy (TEM) (Fig. 1b) and UV-Vis spectrophotometry. The LSPR absorption peak at 528 nm confirms the presence of Au NPs (Fig. 1c) of approximately 20 nm diameter (Fig. 1b). Prior to experiments with biological cells, the nanoelectrodes were tested using an electrochemical cell having 0.1 M phosphate buffer with 0.05 M EDTA (Fig. 2a). Photocurrents were observed during the illumination of the Au nanoelectrode with 532 nm green light, while there were no photocurrents for uncoated glass micropipettes (Fig. 2b). Lowe *et al*.^20^ demonstrated similar photocurrents for Au NP-coated indium tin oxide electrodes and attributed it to the temperature rise at the nanoelectrode-electrolyte interface by plasmonic excitation of Au nanoparticles. The temperature rise at the interface led to alteration of the open circuit voltage of the nanoelectrode with respect to the reference electrode, resulting in photocurrents. These photocurrents vary linearly with applied voltage (Fig. 2d). Photocurrents were also recorded when an extracellular solution (ECS) having a composition of 140 mM NaCl, 5 mM KCl, 1 mM MgCl_2_, 2 mM CaCl_2_, 10 mM HEPES at pH 7.4 maintained with NaOH, was used as the electrolyte instead of phosphate buffer (Fig. 2c) which also exhibited a linear relation with applied voltage (Fig. 2e). Due to SPR, gold nanoparticles are known to affect the emission spectra of fluorophores in its vicinity via energy transfer between the two and are utilized in many fluorescence-based applications like molecular imaging, and sensing^21–26^. Aslan *et al*.^21^ studied the effect of Au NP size on emission spectra of FITC-labeled human serum albumin. We adapted the same methods for testing the nanoelectrodes for fluorescence quenching by the Au NPs^21, 22^. FITC-labeled human serum albumin (HSA) was attached to the Au NP-coated micropipette by incubating the nanoelectrode with a 10 μM solution of the fluorophore. A 488 nm laser source was used to excite FITC-HSA and the emission spectrum the wavelength of ~520 nm was observed under an Olympus BX61W1 upright microscope. Fluorescence quenching was observed in the presence of Au NPs (Supplementary Fig. S1b,c) in comparison to uncoated glass micropipette tips (Supplementary Fig. S1a).

**Figure 3:**
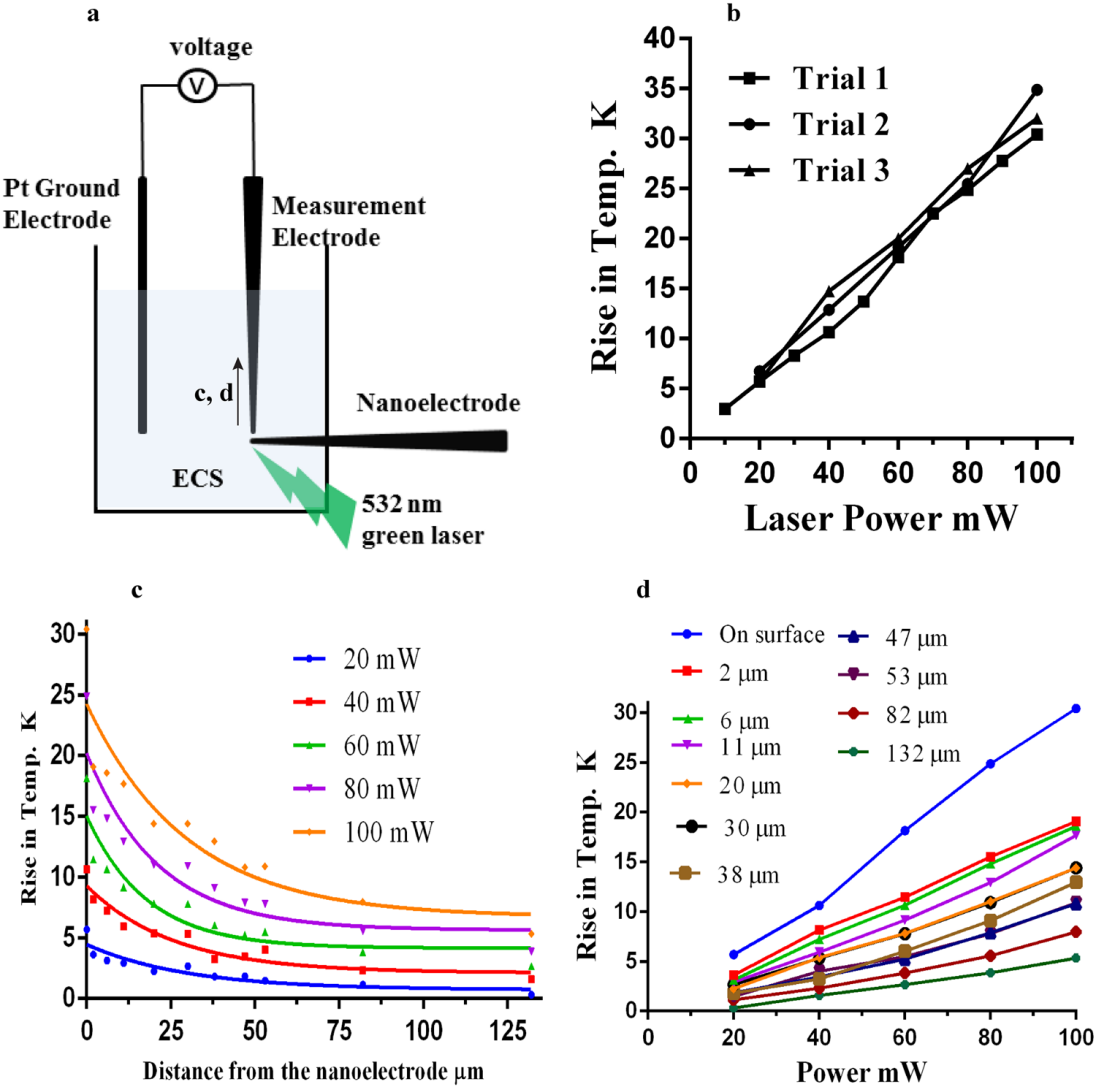
Localized temperature rise measurements. (**a**) Schematic diagram of the plasmonic temperature rise measurements on/near the surface of the nanoelectrode. The experiments were performed in a 35 mm Petri dish filled with extracellular solution (ECS). The measurement electrode was a glass micropipette filled with ECS. A change in resistance was measured in response to a 532 nm green laser focused on the nanoeletrode tip. The resistance vs temperature function was utilized to get the temperature values. (**b**) The rise in temperature on the surface of the nanoelectrode has a linear relation with laser power. (**c**) The temperature decays exponentially with the distance away from the nanoelectrode. The different colors indicate the different laser power settings. The results were fitted to the equation: T = (T_o_ − C)*exp (−kx) + C [parameter values are provided in Supplementary Table 1]. (**d**) Plasmonic temperature rise versus laser power at different distances away from the nanoelectrode.

The ECS solution was heated to approximately 55 °C, then, allowed to cool down to room temperature. The temperature was monitored using a thermistor. The resistance of a patch pipette filled with the same ECS was recorded as the solution cooled down. Supplementary Fig. S2c represents the log (R) vs 1/temperature calibration curve, fitted to a straight line [log R = 868.5 × (1/T) + 3760; R^2^ = 0.9027]. For plasmonic temperature measurement, a patch pipette was placed just next to the nanoelectrode and 20 mV voltage pulses were applied to the patch pipette. The change in resistance was measured in response to stimulation with a 100 mW, 532 nm green laser shined on the tip of the nanoelectrode (Supplementary Fig. S2a). There was no change in resistance for the control condition (no nanoelectrode) (Supplementary Fig. S2b). This resistance change was converted into temperature changes using the calibration curve (Supplementary Fig. S2c). Notice that the plasmonic temperature increases linearly with the laser power (Fig. 3b,d) and decreases exponentially with the distance away from the nanoelectrode (Fig. 3c). A 0.3 to 0.4 ( ± 0.1) °C rise was observed when measured with a thermal camera (Supplementary Fig. S3). These experiments confirm the plasmonic temperature rise around the nanoelectrode when illuminated with green visible laser.

### Excitatory Neural Responses

**Figure 4:**
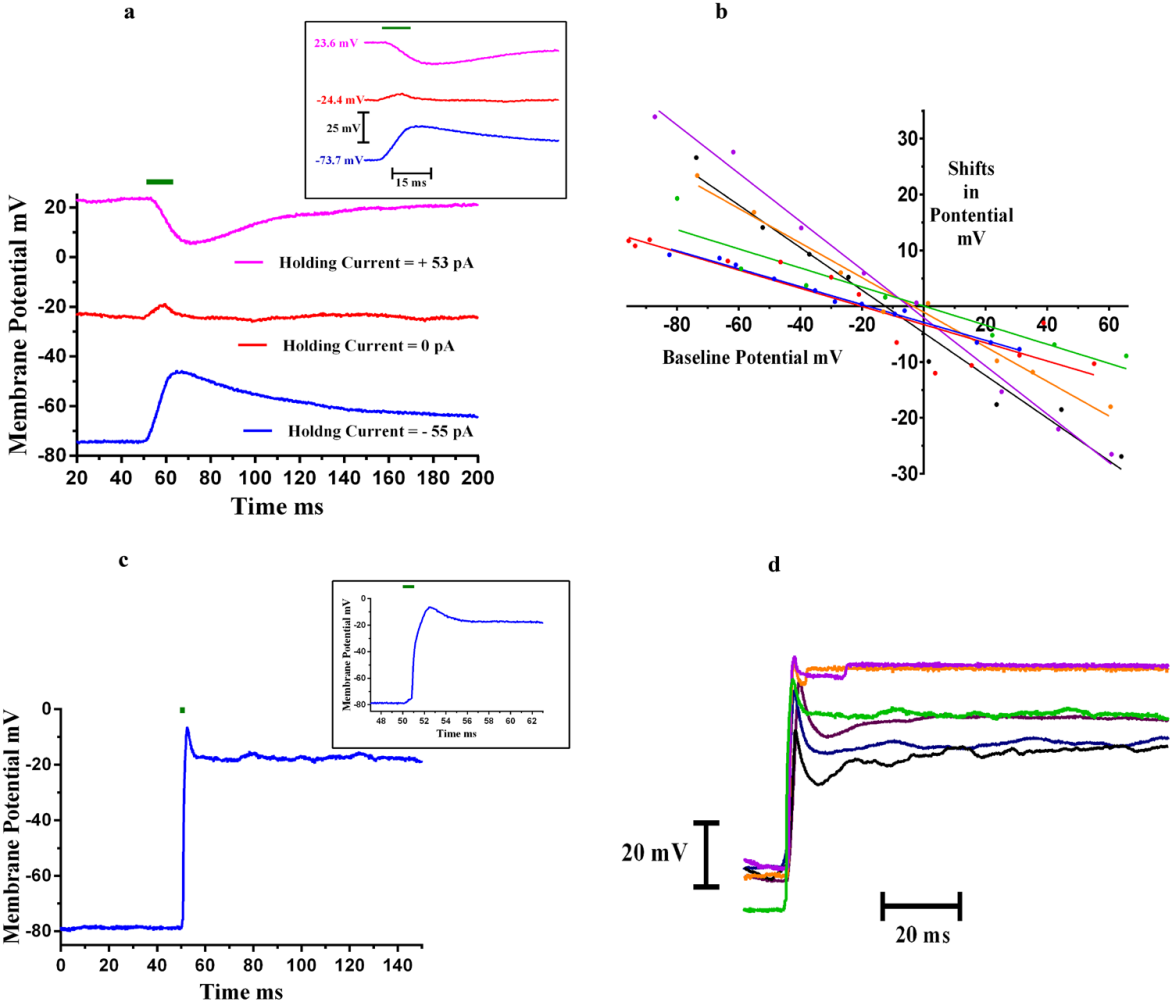
Activation of SH-SY5Y Neural Cells – Whole Cell Current Clamp Recording. (**a**) When a 532 nm green laser (denoted by the green bar), 10 ms pulse, having a power of 100 mW was shined on the tip of the nanoelectrode, a change in cell membrane potential was observed. The inset shows the onset response in more detail (faster time scale). The recordings were done in whole cell current clamp configuration. The figure shows shift in evoked potentials at three different baseline potentials; −73.7 mV (applied holding current = −55 pA), −24.4 mV (applied holding current = 0 pA) and +23.6 mV (applied holding current +53 pA). (**b**) The change in evoked potential varied with membrane potential; positive for −30 mV or less, and negative for 20 mV or more. Shifts in evoked potential vs baseline membrane potential for six different cells are shown, each represented by a different colour. (**c**) For 1–5 ms laser pulses (75–120 mW laser power), action potentials were recorded from the SH-SY5Y neural cells. The figure shows a representative action potential recorded from an SH-SY5Y cell for a 1 ms laser pulse at 100 mW laser power. The inset shows the zoomed portion of the onset response of the same. The cell membrane baseline potential was −78.5 mV (applied holding current =− 35 pA). (**d**) Action potentials recorded from the six different neural cells, each cell shown in a different colour. All the experiments were done in whole current clamp mode using an electrophysiological patch clamp system.

The experiments were done with SH-SY5Y cells in the standard whole cell current clamp configuration using a patch clamp system. The holding current was adjusted to set the membrane potential to a particular baseline value. Light was focused on the electrode via an optical fiber and membrane conductance were recorded using a patch electrode (Supplementary Fig. S4a). The nanoelectrode was placed within 2 µm of the cell membrane. When the nanoelectrode was illuminated with a 532 nm, 100 mW laser for 10 ms or more, a shift in the cell membrane potential was observed, and changed as a function of the cell holding potential (Fig. 4a). Specifically, for −30 mV or less, there were positive shifts in the membrane potential and as the membrane potential approached zero, the magnitude of potential shifts decreased and became negative for positive holding potentials. Fig. 4a shows an example for a 10 ms, 100 mW laser pulse. It can be seen that at a cell membrane potential of −73.7 mV (applied holding current = −55 pA), there was positive shift in the evoked membrane potential – blue curve, and, for a positive cell potential of +23.6 mV (holding current = +53 pA), there was negative shift – pink curve. The SH-SY5Y cells fired typical action potentials in response to electrical stimulation before and after the optical simulation was applied to each cell. Fig. 4b shows the paired relations between shifts in evoked potential in response to laser-nanostimulation and the baseline potential (determined by applied current) for six different cells, each cell shown by a different colour. The positive shifts in evoked membrane potential indicates partial depolarization and negative shifts indicates partial hyperpolarization. We observed that the magnitude of plasmonic jumps increased with an increase in laser power (Supplementary Fig. S4b) and also with increase in laser pulse duration (10–50 ms) (Supplementary Fig. S4c). Similar shifts in evoked potential jumps were observed for neonatal rat cardiomyocytes (Supplementary Fig. S4d). For laser pulse widths of 1–5 ms, action potentials were recorded from the cells. Fig. 4c shows action potential recordings for a representative SH-SY5Y cell when the nanoelectrode was illuminated with a 1 ms 532 nm green laser pulse with 100 mW power. Fig. 4d shows these optically stimulated action potential recordings from six different cells. To induce optical action potentials, we found that laser power should be in the 75 mW–120 mW range. At lower powers below 60 mW, no cellular responses were observed. There was no difference between electrically-evoked action potentials prior to and after the optical stimulation in response to electrical current pulses (Supplementary Fig. S4e). For control experiments (laser stimulation alone - without nanoeletrode −10 ms, 100 mW), no evoked potentials were observed (Supplementary Fig. S6). Following stimulation the membrane potential slowly returns to pre-stimulus values over a time span of seconds and up to a minute. Carvalho-de-Souza *et al*.^14^ also reported the similar observations for stimulation of dorsal root ganglion (DRG) neurons using functionalized Au NPs attached to cell membrane. They have attributed it to transient membrane alteration due to optical stimulation.

### Inhibitory Cell Responses

**Figure 5:**
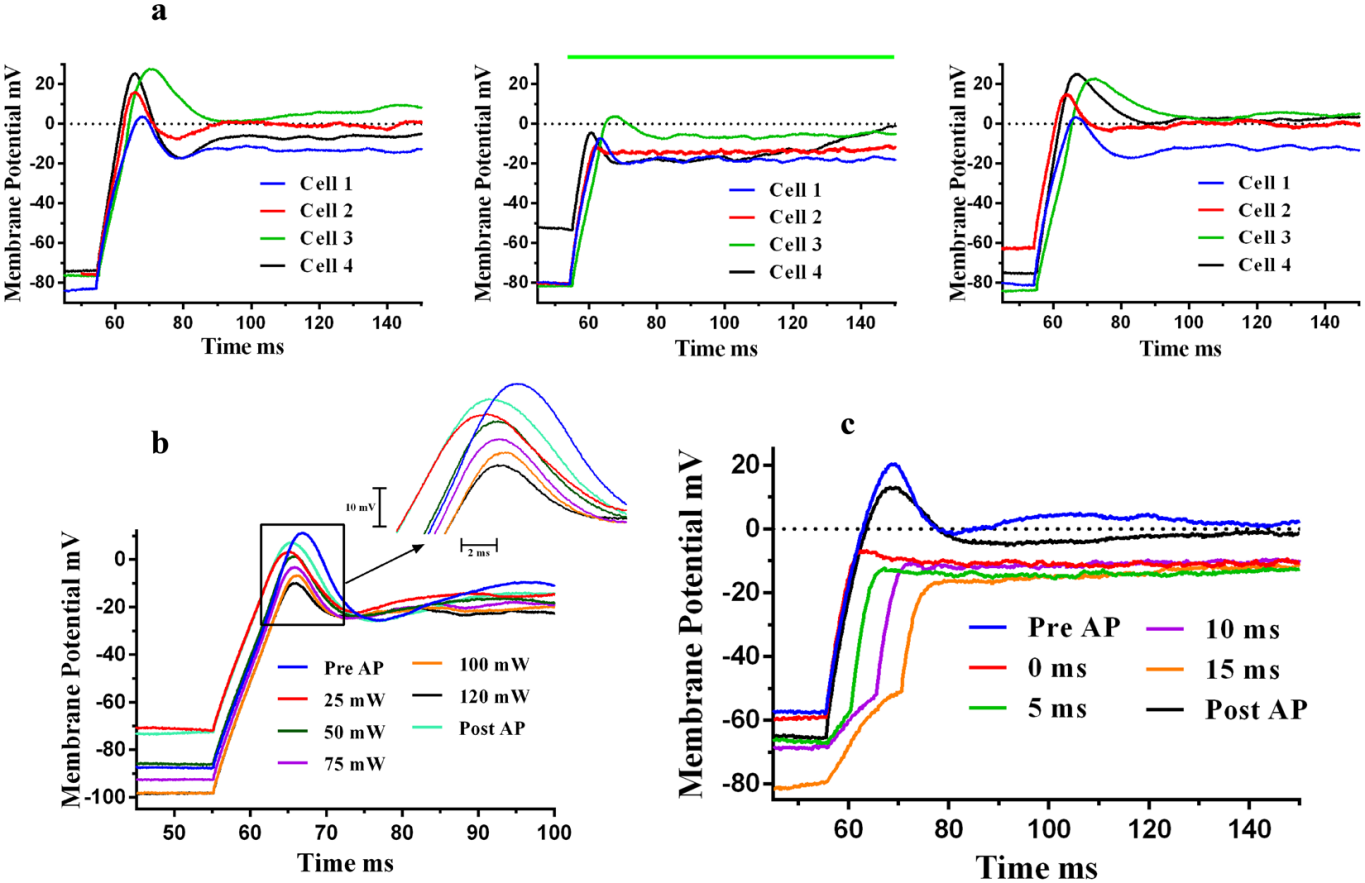
Inhibition of action potentials of SH-SY5Y neural cells - Whole Cell Current Clamp Recording. When laser pulses were superimposed on the responses to electric current pulses, a reduction in magnitude of action potentials was observed. (**a**) The figures show responses for four different cells. Left: Action potentials (Pre-AP) recorded when cells were stimulated with electric current pulses, (180 pA, 300 ms) – control conditions. Middle: A 300 ms, 120 mW, 532 nm green laser pulse (shown by green bar) was superimposed on the electric current pulse (180 pA, 300 ms), cell shows a reduction in the amplitude of action potential. Right: Action potential recorded post laser experiments, returns to the original value. The temp. rise of around 20 K was experienced by ~5–10% cell area. (**b**) The inhibition of action potential is affected by laser power. As laser power increases, inhibition becomes more prominent - AP peak decreases with laser power. The pulse width was 300 ms. (**c**) The inhibition of action potential is more prominent when the laser pulse led the electrical pulse by a few milliseconds as shown in a representative cell.

**Figure 6:**
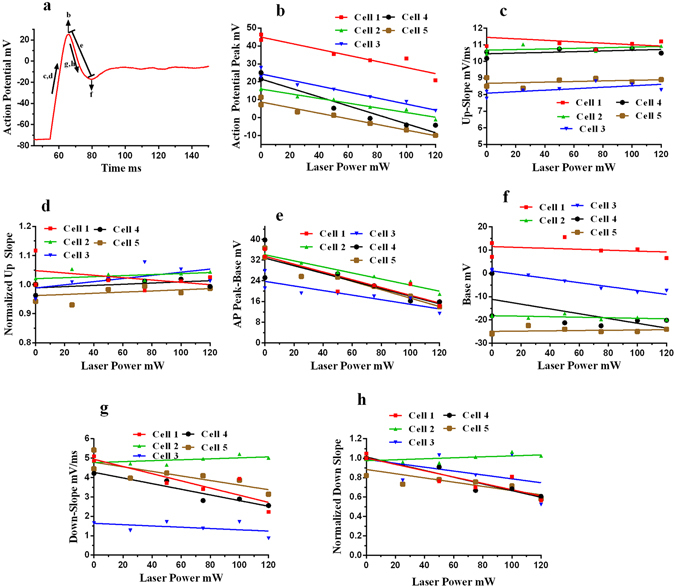
Analysis of inhibition experiments. (**a**) A representative figure indicating the various analyses done for inhibition experiments as function of laser power, shown in subsequent parts. (**b**) Action potential peak decreases as the laser power increases, shown for five different cells. (**c**) The rate of rise of action potential (mV/ms) remains unaffected by the laser power as shown in up-slope vs laser power curves for five different cells. (**d**) All the up-slope values were normalized with initial up-slope (Pre-AP up-slope) which shows same trend of having no change with laser power like absolute values in (**c**). (**e**) The difference between AP peak and base value (first minima after peak) decreases with laser, because of the decrease in peak values of AP (**b**). (**f**) The base value (first minima) remains the same, doesn’t vary with laser power. (**g**,**h**) The down-slope and normalized downslope (downslopes normalized with downslope of pre-AP down-slope) decrease with laser power. The points with zero laser power belongs to electrical stimulations alone, control condition.

Like activation experiments, inhibition experiments were also performed in whole cell current clamp configuration. The laser pulses were delivered using the optical fiber and the current pulses were delivered via patch pipette (Supplementary Fig. S4a). Action potentials were evoked in SH-SY5Y cells using 180 pA, 300 ms current pulses. During simultaneous presentation of optical pulses (300 ms, 120 mW), we observed decreases in the magnitude and rate of the action potentials as compared to the control conditions, i.e., electrical stimulation alone. Action potentials were recorded before and after the optical stimulation experiments (Fig. 5a) and we found no deleterious effects. Similar inhibition of action potential magnitude was observed for spontaneous beating neonatal rat cardiomyocytes (Supplementary Fig. S5). As laser power increased, the inhibition becomes more prominent (Fig. 5b). The inhibition was most prominent when the laser pulse led the electric current pulse by a few milliseconds (5–15 ms) (Fig. 5c). Typically for action potentials, depolarization happens predominantly because of Na^+^ influx and repolarization happens mainly because of K^+^ efflux. Different data analyses were carried out with respect to laser power for depolarization and repolarization phases of the action potentials as shown in Fig. 6a: action potential peaks (AP peaks), up-slope of the AP (rate of the membrane potential rise from baseline potential to peak value: rate of depolarization), normalized up-slope (all the up-slope values are normalized by dividing each value with the initial value of the up-slope for electrical action potentials, recorded before the optical stimulation experiment), differences between the initial peak value and first minimum of the action potential (AP Peak – Base), base value (first minimum after peak value), the down-slope of the AP (rate of membrane potential fall from the peak value to base value: rate of depolarization) and normalized downslope of AP (like up-slopes, downslopes were also normalized with initial value of downslope for electrical action potentials, recorded before the optical stimulation experiment). The AP peak value decreased as laser power increased (Fig. 6b). The up-slope (absolute and normalized) depolarization rate, remained unaffected by the laser power (Fig. 6c,d) while the down-slope (absolute and normalized) repolarization rate, decreases with increases in laser power (Fig. 6g,h). The mean data also confirms the same trends with laser power (Supplementary Fig. S7). As depolarization is mostly governed by Na^+^ influx and repolarization is governed by K^+^ efflux, it is possible that during inhibition, the degree of K^+^ efflux was affected more than the rate of Na^+^ influx. The minimum after the AP peak, i.e., base value did not change with increases in laser power (Fig. 6f), and the difference between the peak & base values decreased with laser power (Fig. 6e).

## Discussion

**Figure 7:**
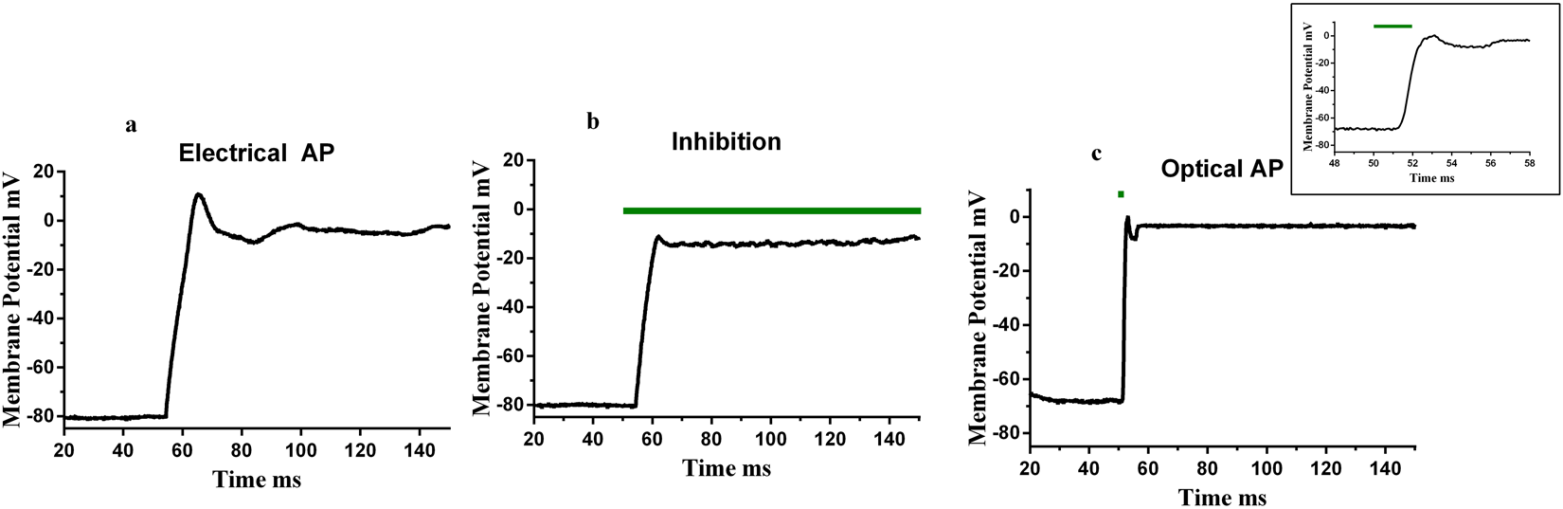
Optical inhibitory and excitatory response – Whole Cell Current Clamp Recording. (**a**) Action potential of a representative SH-SY5Y cell in response to 180 pA, 300 ms electrical pulse. Subsequently, inhibition (120 mW, 532 nm green laser pulse was superimposed on 180 pA electric current pulse) and activation (2ms, 120 mW laser pulse) in response to laser stimulation were recorded. Part b and c shows the inhibition and activation recordings respectively, inset of part c shows the long time scales of the optical action potential.

We have demonstrated for the first time that Au nanoelectrodes in combination with a 532 nm green laser can stimulate and inhibit neural and cardiac action potentials. This is the first report showing *both* inhibition as well as activation of action potentials using Au NPs coated micropipettes and a visible-light laser. We demonstrated these phenomena for two cell models – the human neuroblastoma cell line and neonatal cardiomyocytes. In addition, we were able to record both inhibitory and excitatory responses from the same cells, depending on the properties of laser stimulation (e.g., Fig. 7). We found that to elicit action potentials, the laser timing is a critical parameter. In the present investigation, the cells fired action potentials for short laser pulses with 1–5 ms durations (Fig. 4c and d). For longer pulses (10 ms or higher), though there was a shift in the evoked cell potential in response to the laser stimulation, no excitatory action potentials were elicited (Figs 4a and b, Supplementary S4b, c & d). In the case of opto-electrical stimulation (laser pulse with electric current pulse for stimulation – timing 300 ms) of SH-SY5Y cells, we discovered that inhibition of action potentials happens (Fig. 5a and b), an observation consistent with our experiments with spontaneous beating cardiomyocytes, i.e., decreases in the magnitude of action potentials were elicited (laser pulse timing = 10 s) (Supplementary Fig. S5).

Certain aspects of the discoveries here are consistent with recent reports in the literature. A few previous studies report neural excitatory activation with pulse widths of a few milliseconds^14, 15^, and for inhibition, the laser durations were for longer time periods (tens to hundreds of milliseconds)^19, 27^. Specifically, Yoo *et al*.^19^ reported inhibition of neural activity for hippocampal tissue slices when stimulated with gold nanorods and a 785 nm near-IR laser. The laser pulse durations were from several seconds to a few minutes. We observed similar inhibition of neural activity in our plasmonic stimulation experiments but only for longer (300 ms) laser pulse durations. Similarly, Eom *et al*.^15^ showed neural activity enhancement utilizing IR stimulation for rat sciatic nerve when the nerve was stimulated in the presence of Au nanorods, as compared to the control condition (laser with no Au nanorods). The laser pulse duration was less than 3 milliseconds, a similar duration for which we observed neural activation (1–5 ms) in the present study. What is exciting about the discoveries we present here is the possibility of stimulating using electrodes and visible light, leading to translation to prosthetics such as cochlear implant electrodes constructed of suitable polymer materials.

To make significant progress towards clinical applications, it is important to move towards understanding the biological mechanisms for light-activated Au NPs neural stimulation. Metal nanoparticles like Au have strong surface interactions with electromagnetic fields – light, due to availability of free electrons in their conduction bands. For small size particles (<20 nm) nearly all energy is absorbed and converted into heat^28, 29^. Since we used particles of approximately 20 nm, it is likely that non-radiative decay (localized plasmonic heating) plays a critical role in the neural stimulation reported here. In addition, there are recent reports that IR lasers can invoke *in vivo* responses^30–34^. A likely mechanism for IR stimulation is a temperature rise due to photothermal interactions with cell membrane capacitance properties^35, 36^. In particular, Shapiro *et al*.^35^ showed that the absorption of IR laser energy, causing a temperature rise, resultant changes in cell membrane capacitance are sufficient for cell depolarization. The artificial bilayer was used as the model of the cell membrane, and capacitance changes were evaluated in response to IR stimulation. So, it is very likely that localized plasmonic heating due to Au nanoelectrode interactions with visible light alters the cell capacitance. Carvalho-de-Souza *et al*.^14^ also suggested a similar mechanism to explain how conjugated Au NPs enables action potential activation in dorsal root ganglion neurons. Like Shapiro *et al*.^35^, an artificial bilayer was considered as a representative model of cell membrane capacitance changes. In sum, initial reports suggest that membrane capacitance change is the driving force for action potential generation in response to short thermal pulses. Apart from the transient capacitance change mechanism, there are indirect indications that heat sensitive channels could also play a role in depolarization^37^.

One more possible physical effect of the plasmonic stimulation is alteration of cell membrane properties by the thermal pulse. This could potentially involve transient nanopore formation in the cell membrane, facilitating a nonspecific ion permeability increase, altering the cell potential and activating voltage-gated ion channels, as suggested by Beier and colleagues^38–42^. They reported the influx of Ca^2+^ ions due to activation of intracellular pathways, can also affect cell membrane fluidity when cells were exposed to millisecond-long infrared laser pulses^38, 41^. Eom *et al*.^43^ reported that astrocytes released intracellular Ca^2+^ in response to Au nanorods stimulated with IR light. Earlier studies reported that inhibition due to thermal pulses is likely mediated by potassium channels^19, 27, 44^. For example, Yoo *et al*.^19^ reported the inhibition of hippocampal neurons in response to illumination of gold nanorods with 785 nm near IR laser. The inhibition was attributed to the involvement of a thermo-sensitive potassium channel – TREK-1. No neural suppression was observed in presence of fluoxetine – a TREK-1 blocker. Rabbitt *et al*.^44^ demonstrated IR stimulation of type II vestibular hair cells *ex vivo* from mice and afferent neurons *in vivo* from chinchillas. The inhibition responses were thought to be governed by large conductance Ca^2+^ activated potassium channels, so called BK channels. The application of iberiotoxin (IBTX) – a selective blocker for BK channels, eliminated this inhibitory response. Relevant to understanding cellular mechanisms for the present study, SH-SY5Y cells have both TREK-1 and BK channels^45–47^. As evident from the above-mentioned studies, there are multiple effects of thermal pulses that could govern the underlying mechanisms of thermal stimulation. Further studies are needed to investigate/understand detailed mechanisms. Along with considering the Hodgkin-Huxley models at macro levels, it will be worthwhile to explore individual channel kinetics and membrane properties at nano-levels with the help of techniques like molecular dynamics simulations.

Nanoparticle-assisted neural modulation is still in the early research stages. Most studies are focused on *in vitro* proof of concept^14–18, 48, 49^, with very few *in vivo* experiments^12, 16^. *In-vivo* methods involving nanoparticles raise the issues of toxicity, reliability and bio-compatibility^12, 15, 16^. Our method of using nanoelectrodes (micro-substrates coated with nanomaterials) which provide a physical buffer between the particles and neural tissue, and optical fibers for delivering laser light locally holds much promise for addressing many of these problems. In-fact, Tran *et al*.^50^ reported that surface-bound Au NPs reduced the cytotoxicity as compared to unbound nanoparticles. They have also used γ-(aminopropyl) triethoxysilane to bind particles to silica surfaces. With a nanoelectrode or similar probe, there is no need to attach nanoparticles to the neurons or alteration of cells at the stimulation interface. In addition, as we reported here, nanoelectrodes can be easily validated for plasmonic responses independently using various methods, such as light-induced photocurrents (few 100 s of nA), fluorescence quenching (emission spectra around 520 nm), plasmonic temperature jumps (pipette resistance method and infrared thermograms). Our nanoelectrode design can be easily adapted for *in vivo* applications by using nanomaterial-coated biocompatible materials instead of borosilicate glass. There are biocompatible materials which can be used for these prosthetic devices, e.g., polymides, silicon polymers and polymers like polyethylene, polytetrafluoroethylene and polydimethyl siloxane^51, 52^. More specifically, there are biocompatible polymers like poly (methyl methacrylate) - (PMMA) and poly (dimethylsiloxane) – (PDMS) which are known to work with silane linkers^53–55^. Subsequently, functionalized surfaces can be used to develop Au NPs coated microelectrodes via dip coating methods^56^. It is even possible to use the glass to develop initial stimulating microelectrodes^57^. Implantable silicone would be a good carrier for microelectrodes as it is currently used in many medical applications like cochlear implants, blood vessel, heart valves and prosthetic outer ears^52, 58, 59^. Light can be delivered via commercially available optical fibers or waveguides. Hence, based on the stimulation results of this report, it is possible to design biocompatible prosthetic devices.

Our future studies will be focused on developing more robust plasmonic stimulation systems by better understanding the biological mechanisms involved, and teasing apart the interactions of nanomaterial/laser parameters for controlling differential neural and cardiac stimulation responses. We will also explore the *in vivo* applications of plasmonic stimulation with an eye towards the development, initially, of plasmonic-based cochlear implants for deaf people. One of our proposed designs involves a neural cuff or flexible substrate that will employ nanomaterial-coated micro-beads along with focal light-fiber sources. The substrate or cuff has the potential for carrying dozens of electrodes as compared to a maximum of up to 22 electrodes found in current cochlear implants^60^; with the goal that the new laser stimulation device would stimulate much more discrete groups of ANFs. A more direct method would be adaptation of the currently used electrical stimulation cochlear electrode array with optical fibers replacing the electrical electrodes. In an easy modification of the design, one could structure the electrode to locate polymer-electrode material coated Au NPs very close to the basilar membrane, affording extremely specific stimulation, without thermal damage by the visible light utilized.

Overall, the present report provides fundamental new evidence to support the pursuit of plasmonic stimulation as an alternative approach to conventional electrical neuromodulation, or other emerging modalities, such as IR stimulation. Because the novel technology of the current investigation has capabilities to elicit both inhibitory and excitatory responses, utilizing different stimulation parameters, this type of plasmonic stimulation can be a promising alternative for electrical stimulation paradigms in biological prosthetic implants. As emphasized above, the major disadvantage with IR stimulation is that, along with the target neurons, the IR laser heats up the surrounding tissue as well, which can cause thermal damage and/or unwanted excessive stimulation. By combining visible light, which is minimally absorbed by surrounding tissue and aqueous solutions, with an engineered nanomaterial, we have the potential to achieve a unique, highly localized, yet temporally precise, delivery of energy to the target cells to manipulate their bioelectrical behavior.

## Methods

### Fabrication of Nanoelectrodes for Cell Stimulation

Au NPs were coated onto glass micropipettes to fabricate the nanoelectrodes for cellular stimulation. Glass micropipettes were pulled using P-97 micropipette puller (Sutter Instruments, Novato, CA). The micropipettes were washed with detergent while heating at 60 °C for 10–15 min, then, washed thoroughly with distilled (DI) water. The micropipettes were then cleaned with a mixture of HCl and methanol (1:1 volume/volume) and washed with DI water, and heated in an oven at 60°C. Next, the micropipettes were immersed in 10% volume/volume solution of γ-(aminopropyl) triethoxysilane (Sigma-Aldrich, St. Louis, MO; APTES, ≥98%) in anhydrous ethanol for 15–20 min. Finally, the pipette tips were washed with ethanol extensively 5x before heating at 120 °C for 3 h. The tips functionalized with amino silane linkers were immersed in Au NPs for 24 h^56^. The final size of micropipettes was 1–2 µm. Au NPs were synthesized using a liquid phase bottom-up approach in which an Au salt solution was reduced with trisodium citrate dihydrate solution. The glassware used for synthesis of Au NPs was thoroughly cleaned with DI water, followed by another thorough cleaning with ethanol. A 20 ml solution of 1 mM gold (III) chloride trihydrate (HAuCl_4_.3H_2_O) (Sigma-Aldrich, ≥99.9%) was boiled on a hot plate in a beaker with continuous stirring. Then, 2–3 ml of a 1% solution of trisodium citrate was added to the boiling HAuCl_4_ solution. The solution was boiled until it turned deep red in color, showing the presence of Au NPs^56^. Au NPs were characterized using a UV-Vis spectrophotometer (Perkin Elmer, Waltham, MA; Lambda 35 spectrophotometer) and a transmission electron microscope (FEI Morgagni, Hillsboro, OR). The nanoelectrodes were characterized using a scanning electron microscope (Hitachi SU70 SEM, Krefeld, Germany).

### Testing of the Nanoelectrodes

Before proceeding to the *in vitro* experiments with electrically excitable biological cells (SH-SY5Y and neonatal cardiomyocytes), light-induced photocurrents were recorded when the nanoelectrodes were illuminated with a 532 nm 100 mW green laser (Laser Quantum, Santa Clara, CA; gem 532 high spec OEM laser). The photocurrents were measured using a Gamry reference 600^TM^ potentiostat and recorded using Gamry Instruments Framework software (Gamry Instruments, Warminster, PA). The instrument was calibrated with a UDC4 dummy cell periodically. A 0.1 M phosphate buffer solution containing 0.05 M EDTA was used as the electrolyte^20^. The nanoelectrode (micropipette coated with Au NPs) and a Pt electrode were used as the two electrode of the electrochemical cell whereas saturated calomel electrode served as the reference electrode. The micropipette (not coated with Au NPs) was used for the control condition. These tests were also carried out using an extracellular solution (ECS) as the electrolyte. The nanoelectrodes were tested for fluorescence quenching by attaching FITC-labeled human serum albumin (HSA) to the nanoelectrode surface and illuminating it with 488 nm blue light under a fluorescence microscope: Olympus BX61W1 upright microscope.

### Measurement of Localized Temperature Changes

Plasmonic temperature elevations on/near the surface of the nanoelectrode were measured using an indirect, pipette resistance method previously developed to measure local temperature changes for infrared stimulation of biological cells^35, 61^. A micropipette having a resistance of 5–8 MΩ was filled with ECS and placed near the surface of the nanoelectrode. A 532 nm 100 mW green laser was focused on the tip of the nanoelectrode with the help of an optical fiber having an inside diameter of 50 µm. The experiments were done using a patch clamp system (Multiclamp 700B amplifier and 1440 A data acquisition Molecular Devices, Sunnyvale, CA). A voltage of 20 mV was applied to the circuit and resistance changes were observed when the laser was focused on the nanoelectrode. Control experiments were performed in the absence of the nanoelectrode. The resistance (log R) versus temperature (1/temp. [K]) calibration curves were obtained in response to a 20 mV voltage pulse by allowing the hot ECS solution (approximately 55 °C starting point) to cool down to room temperature in a Petri dish. The temperature was continuously monitored using a thermistor (monitor thermistor of temperature controller TC-324 unit from Warner Instruments, Hamden, CT). As temperature goes down, corresponding resistance was measured. Temperature variation was measured as functions of laser power and distance from the nanoelectrode. Plasmonic temperature increases were also confirmed using an infrared thermal camera (FLIR T420 thermal camera with detector spectral range 7.5 to 13.0 µm).

### Cell Culture

#### Neuroblastoma Cells

Neuroblastoma, SH-SY5Y (ATCC^®^ CRL-2266) can be differentiated into neurons in presence of retinoic acid. The cell lines were cultured in a mixture of DMEM and F12 (1:1 volume/volume) media containing 10% FBS and 1% penstrep. Cells were cultured at 37 °C with 5% CO_2_. After 48 h of plating, the media was replaced with neurobasal media containing B27, GlutaMAX and penstrep as the supplement. 10–20 µM of all-trans-retinoic acid (ATRA) was added into the media to facilitate the differentiation. The media was changed every 48–72 hr^62, 63^.

#### Neonatal Cardiomyocytes

The heart tissue was provided by the laboratory of Prof. Cheryl Kirstein, Department of Psychology, University of South Florida, Tampa, FL. Neonatal cardiomyocytes were obtained from 2–3 day old Sprague Dawley rat pups. 8–10 pups were decapitated and their hearts were removed. The hearts were transferred to ice cold PBS containing 20 mM glucose. The atria were removed using a small scissors to expose the ventricle cardiomyocytes, which were subsequently, minced into small pieces. For digestion, the collagenase solution was prepared by adding collagenase type II (1 mg/ml) and 1% bovine serum albumin (BSA) into phosphate buffered saline (PBS) having 20 mM glucose. The minced ventricle parts were digested using collagenase solution. The cells were cultured in M199 media, having 5% FBS, 10% horse serum and 0.1% penstrep. The cells were incubated at 37 °C with 5% CO_2_. The cardiomyocytes showed spontaneous beating in days 1–2^64^. All the animal protocols were approved by University of South Florida Institutional Animal Care and Use Committee (IACUC) and are consistent with US Federal and NIH guidelines.

#### *In vitro* Cell Stimulation

*In vitro* stimulation experiments were done using a patch clamp system in the whole cell current clamp configuration. These physiological experiments were done using a Multiclamp 700B amplifier and Digidata 1440 A data acquisition system (Molecular Devices, Sunnyvale, CA). Experiments were carried out at room temperature. Patch pipettes were pulled using a P-97 micropipette puller (Sutter Instruments, Novato, CA) and their capacitance was corrected using Multiclamp software. After the giga-Ω seal on the cell membrane with the pipette, the whole cell configuration was achieved by applying suction pressure to the patch pipette using a 1 ml syringe. Subsequently, the amplifier (Multiclamp 700B, Molecular Devices, Sunnyvale, CA) was switched to the current clamp mode. Then, the nanoelectrode was placed just next (within 2 µm) to the patch cell. A 532 nm green laser was focused on the tip of the nanoelectrode using an optical fiber having inside diameter of 50 µm. Two 532 nm lasers were used for this study – A Gem 532 nm green laser from Laser Quantum (Santa Clara, CA) and OBIS 532 nm laser from Coherent (Santa Clara, CA). The lasers were controlled using a function generator (AFG 320 Sony Tektronix, Beaverton, OR). The power was varied by altering the power of input voltages to the laser and the lasers were calibrated on a monthly basis using a power meter (Coherent, Santa Clara, CA; FieldMAX light power meter). The cell membrane potential was adjusted by applying a holding under current clamp mode. In this way, we sought to determine the effects of laser stimulation on evoked membrane potentials. For 10 ms, 100 mW laser pulse stimulation experiments (Fig. 4a and b), a series of holding currents were applied to do the paired analysis of evoked potentials at different baseline membrane potentials. For inhibition experiments, laser pulses were applied simultaneously with electrical current pulses under current clamp mode and membrane potential responses were recorded. Data acquisition and analysis were performed with pClamp & Clampfit (Molecular Devices, Sunnyvale, CA) and GraphPad Prism (La Jolla, CA) software packages.

For SH-SY5Y cells, the extracellular solution (ECS) composition was 125 mM NaCl, 4 mM KCl, 2 mM CaCl_2_, 1.2 mM MgSO_4_, 10 mM glucose and 10 mM HEPES, with pH maintained at 7.4 via NaOH. The intracellular solution (ICS) composition was 140 mM KCl,4 mM NaCl, 0.02 mM CaCl_2_, 0.8 mM EGTA, 2 mM MgCl_2_, 4 mM Mg-ATP and 10 mM HEPES, with pH maintained at 7.2 via KOH. Patch pipette resistance was 5–7 MΩ^65^.

For neonatal cardiomyocytes, ECS was 140 mM NaCl, 5 mM KCl, 1 mM MgCl_2_, 2 mM CaCl_2_, 10 mM HEPES/pH 7.4 (NaOH); and the ICS was 140 mM KCl, 2 mM MgCl_2_, 1 mM CaCl_2_, 5 mM Mg-ATP, 10 mM NaCl and 10 mM EGTA/pH 7.2 (KOH). Patch pipette resistance was 2–4 MΩ^66^.

## Electronic supplementary material

Supplementary information

## References

[1] Wilson BS (1991). Better speech recognition with cochlear implants. Nature.

[2] Hornig R (2005). A method and technical equipment for an acute human trial to evaluate retinal implant technology. Journal of neural engineering.

[3] Wood MA, Ellenbogen KA (2002). Cardiac pacemakers from the patient’s perspective. Circulation.

[4] Firszt JB, Koch DB, Downing M, Litvak L (2007). Current steering creates additional pitch percepts in adult cochlear implant recipients. Otology & Neurotology.

[5] Limb CJ, Roy AT (2014). Technological, biological, and acoustical constraints to music perception in cochlear implant users. Hearing research.

[6] O’Leary SJ, Richardson RR, McDermott HJ (2009). Principles of design and biological approaches for improving the selectivity of cochlear implant electrodes. Journal of neural engineering.

[7] Bingham, R. *Poor Quality EMG Studies are Rampant and Costly*, www.emgaudit.com (2016).

[8] Shin SS, Dixon CE, Okonkwo DO, Richardson RM (2014). Neurostimulation for traumatic brain injury. Journal of Neurosurgery.

[9] Wang, Y. & Guo, L. Nanomaterial-enabled neural stimulation. *Frontiers in Neuroscience* **10**, doi:10.3389/fnins.2016.00069 (2016). 10.3389/fnins.2016.00069 PMC4779906 27013938

[10] Colombo, E., Feyen, P., Antognazza, M. R., Lanzani, G. & Benfenati, F. Nanoparticles: a challenging vehicle for neural stimulation. *Frontiers in Neuroscience* **10**, doi:10.3389/fnins.2016.00105 (2016). 10.3389/fnins.2016.00105 PMC4803724 27047327

[11] Deisseroth K (2015). Optogenetics: 10 years of microbial opsins in neuroscience. Nat Neurosci.

[12] Chen R, Romero G, Christiansen MG, Mohr A, Anikeeva P (2015). Wireless magnetothermal deep brain stimulation. Science.

[13] Marino A (2015). Piezoelectric Nanoparticle-Assisted Wireless Neuronal Stimulation. ACS Nano.

[14] Carvalho-de-Souza JL (2015). Photosensitivity of neurons enabled by cell-targeted gold nanoparticles. Neuron.

[15] Eom K (2014). Enhanced Infrared Neural Stimulation using Localized Surface Plasmon Resonance of Gold Nanorods. Small.

[16] Li W (2015). Remote modulation of neural activities via near-infrared triggered release of biomolecules. Biomaterials.

[17] Pappas TC (2007). Nanoscale Engineering of a Cellular Interface with Semiconductor Nanoparticle Films for Photoelectric Stimulation of Neurons. Nano Letters.

[18] Yong J (2014). Gold‐Nanorod‐Assisted Near‐Infrared Stimulation of Primary Auditory Neurons. Advanced healthcare materials.

[19] Yoo S, Hong S, Choi Y, Park J-H, Nam Y (2014). Photothermal Inhibition of Neural Activity with Near-Infrared-Sensitive Nanotransducers. ACS Nano.

[20] Lowe LB (2003). Laser-induced temperature jump electrochemistry on gold nanoparticle-coated electrodes. Journal of the American Chemical Society.

[21] Aslan K, Malyn SN, Geddes CD (2007). Metal-enhanced fluorescence from gold surfaces: angular dependent emission. Journal of fluorescence.

[22] Aslan K, Pérez-Luna VH (2004). Quenched emission of fluorescence by ligand functionalized gold nanoparticles. Journal of fluorescence.

[23] Dulkeith E (2002). Fluorescence quenching of dye molecules near gold nanoparticles: radiative and nonradiative effects. Physical review letters.

[24] Dulkeith E (2005). Gold nanoparticles quench fluorescence by phase induced radiative rate suppression. Nano Letters.

[25] Lee S (2008). A Near‐Infrared‐Fluorescence‐Quenched Gold‐Nanoparticle Imaging Probe for *In Vivo* Drug Screening and Protease Activity Determination. Angewandte Chemie.

[26] Schneider G (2006). Distance-dependent fluorescence quenching on gold nanoparticles ensheathed with layer-by-layer assembled polyelectrolytes. Nano letters.

[27] Yoo S, Kim R, Park J-H, Nam Y (2016). Electro-optical Neural Platform Integrated with Nanoplasmonic Inhibition Interface. ACS Nano.

[28] Coronado EA, Encina ER, Stefani FD (2011). Optical properties of metallic nanoparticles: manipulating light, heat and forces at the nanoscale. Nanoscale.

[29] Huang X, El-Sayed MA (2010). Gold nanoparticles: Optical properties and implementations in cancer diagnosis and photothermal therapy. Journal of Advanced Research.

[30] Izzo AD, Richter C-P, Jansen ED, Walsh JT (2006). Laser stimulation of the auditory nerve. Lasers in Surgery and Medicine.

[31] Izzo AD (2007). Selectivity of neural stimulation in the auditory system: a comparison of optic and electric stimuli. BIOMEDO.

[32] Izzo AD (2007). Optical parameter variability in laser nerve stimulation: a study of pulse duration, repetition rate, and wavelength. Biomedical Engineering, IEEE Transactions on.

[33] Littlefield PD, Vujanovic I, Mundi J, Matic AI, Richter CP (2010). Laser stimulation of single auditory nerve fibers. The Laryngoscope.

[34] Rajguru SM (2010). Optical cochlear implants: evaluation of surgical approach and laser parameters in cats. Hearing research.

[35] Shapiro MG, Homma K, Villarreal S, Richter C-P, Bezanilla F (2012). Infrared light excites cells by changing their electrical capacitance. Nat Commun.

[36] Wells J (2007). Biophysical Mechanisms of Transient Optical Stimulation of Peripheral Nerve. Biophysical Journal.

[37] Katz EJ, Ilev IK, Krauthamer V, Weinreich D (2010). Excitation of primary afferent neurons by near-infrared light *in vitro*. Neuroreport.

[38] Hope TB, Gleb PT, Joshua DM, Robert JT, Bennett LI (2014). Plasma membrane nanoporation as a possible mechanism behind infrared excitation of cells. Journal of Neural Engineering.

[39] Olsovsky, C. A., Tolstykh, G. P., Ibey, B. L. & Beier, H. T. Origins of intracellular calcium mobilization evoked by infrared laser stimulation. *Proc*. *SPIE 9321, Optical Interactions with Tissue and Cells XXVI*, 93210L (2015).

[40] Roth, C. C., Barnes, R. A., Ibey, B. L., Glickman, R. D. & Beier, H. T. Short infrared (IR) laser pulses can induce nanoporation. *Proc*. *SPIE 9690*, *Clinical and Translational Neurophotonics; Neural Imaging and Sensing; and Optogenetics and Optical Manipulation*, 96901L (2016).

[41] Walsh, A. J., Cantu, J. C., Ibey, B. L. & Beier, H. T. Short Infrared Laser Pulses Increase Cell Membrane Fluidity. *Proc*. *SPIE 10062*, *Optical Interactions with Tissue and Cells XXVIII*, 100620D (2017).

[42] Walsh AJ, Tolstykh GP, Martens S, Ibey BL, Beier HT (2016). Action potential block in neurons by infrared light. Neurophoton.

[43] Eom, K. *et al*. Photothermal activation of astrocyte cells using localized surface plasmon resonance of gold nanorods. *Journal of Biophotonics* **10**, 486–493 (2017). 10.1002/jbio.201600280 28164459

[44] Rabbitt RD (2016). Heat pulse excitability of vestibular hair cells and afferent neurons. Journal of Neurophysiology.

[45] Curci, A., Mele, A., Camerino, G. M., Dinardo, M. M. & Tricarico, D. The large conductance Ca2 + -activated K + (BKCa) channel regulates cell proliferation in SH-SY5Y neuroblastoma cells by activating the staurosporine-sensitive protein kinases. *Frontiers in physiology* **5** (2014). 10.3389/fphys.2014.00476 PMC4260485 25538629

[46] Park JH (2010). High expression of large-conductance Ca 2 + -activated K + channel in the CD133 + subpopulation of SH-SY5Y neuroblastoma cells. Biochemical and biophysical research communications.

[47] Tong L (2014). Activation of K2P channel–TREK1 mediates the neuroprotection induced by sevoflurane preconditioning. British journal of anaesthesia.

[48] Farah N (2013). Holographically patterned activation using photo-absorber induced neural? thermal stimulation. Journal of neural engineering.

[49] Zhao Y, Larimer P, Pressler RT, Strowbridge BW, Burda C (2009). Wireless activation of neurons in brain slices using nanostructured semiconductor photoelectrodes. Angewandte Chemie International Edition.

[50] Tran A, Kaulen C, Simon U, Offenhäusser A, Mayer D (2017). Surface coupling strength of gold nanoparticles affects cytotoxicity towards neurons. Biomaterials Science.

[51] Nag S, Banerjee R (2012). Fundamentals of medical implant materials. ASM handbook.

[52] Stöver, T. & Lenarz, T. Biomaterials in cochlear implants. *GMS current topics in otorhinolaryngology*, *head and neck surgery* **8** (2009). 10.3205/cto000062 PMC3199815 22073103

[53] Kim K, Park SW, Yang SS (2010). The optimization of PDMS-PMMA bonding process using silane primer. BioChip Journal.

[54] Riau AK (2015). Surface modification of PMMA to improve adhesion to corneal substitutes in a synthetic core–skirt keratoprosthesis. ACS applied materials & interfaces.

[55] Sunkara V (2011). Simple room temperature bonding of thermoplastics and poly (dimethylsiloxane). Lab on a Chip.

[56] Nath N, Chilkoti A (2002). A colorimetric gold nanoparticle sensor to interrogate biomolecular interactions in real time on a surface. Analytical chemistry.

[57] Bartels J (2008). Neurotrophic electrode: method of assembly and implantation into human motor speech cortex. Journal of neuroscience methods.

[58] Luria LW (2002). The role of medical grade silicones in surgery and its topical applications. Operative Techniques in Plastic and Reconstructive Surgery.

[59] Ratner, B. D., Hoffman, A. S., Schoen, F. J. & Lemons, J. E. *Biomaterials science: an introduction to materials in medicine*. (Academic press, 2004).

[60] Wilson BS, Dorman MF (2008). Cochlear implants: a remarkable past and a brilliant future. Hearing research.

[61] Yao, J., Liu, B. & Qin, F. Rapid Temperature Jump by Infrared Diode Laser Irradiation for Patch-Clamp Studies. *Biophysical Journal* **96**, 3611–3619, doi:10.1016/j.bpj.2009.02.016. 10.1016/j.bpj.2009.02.016 PMC2711624 19413966

[62] Kovalevich, J. & Langford, D. Considerations for the use of SH-SY5Y neuroblastoma cells in neurobiology. *Neuronal Cell Culture: Methods and Protocols*, 9–21 (2013). 10.1007/978-1-62703-640-5_2 PMC5127451 23975817

[63] Påhlman S, Ruusala A-I, Abrahamsson L, Mattsson ME, Esscher T (1984). Retinoic acid-induced differentiation of cultured human neuroblastoma cells: a comparison with phorbolester-induced differentiation. Cell differentiation.

[64] Salameh, A. & Dhein, S. In *Practical Methods in Cardiovascular Research* (eds Stefan Dhein, Friedrich Wilhelm Mohr and Mario Delmar) 568–576 (Springer Berlin Heidelberg, 2005).

[65] Tosetti P, Taglietti V, Toselli M (1998). Functional Changes in Potassium Conductances of the Human Neuroblastoma Cell Line SH-SY5Y During *In Vitro* Differentiation. Journal of Neurophysiology.

[66] Kang JX, Xiao Y-F, Leaf A (1995). Free, long-chain, polyunsaturated fatty acids reduce membrane electrical excitability in neonatal rat cardiac myocytes. Proceedings of the National Academy of Sciences.

