# Supplementary material for: Nanoparticle-based Plasmonic Transduction for Modulation of Electrically Excitable Cells
Source: Sci Rep. 2017 Aug 10;7:7803. doi: 10.1038/s41598-017-08141-4 (PMC5552804; doi:10.1038/s41598-017-08141-4)
Supplement: Supplementary file 1 — Supplementary information [file 41598_2017_8141_MOESM1_ESM.pdf]

Nanoparticle-based Plasmonic Transduction for Modulation of Electrically Excitable Cells

Parveen Bazard[1,3], Robert D. Frisina[1,2,3], Joseph P. Walton[1,2,3] and Venkat R. Bhethanabotla [*1,3]

1. Department of Chemical and Biomedical Engineering, College of Engineering, University of South Florida, Tampa, FL-33620
2. Department of Communication Sciences and Disorders, College of Behavioral & Community Sciences, University of South Florida, Tampa, FL-33620
3. Global Center of Hearing and Speech Research, University of South Florida, Tampa, FL-33612

* Corresponding Author – bhethana@usf.edu

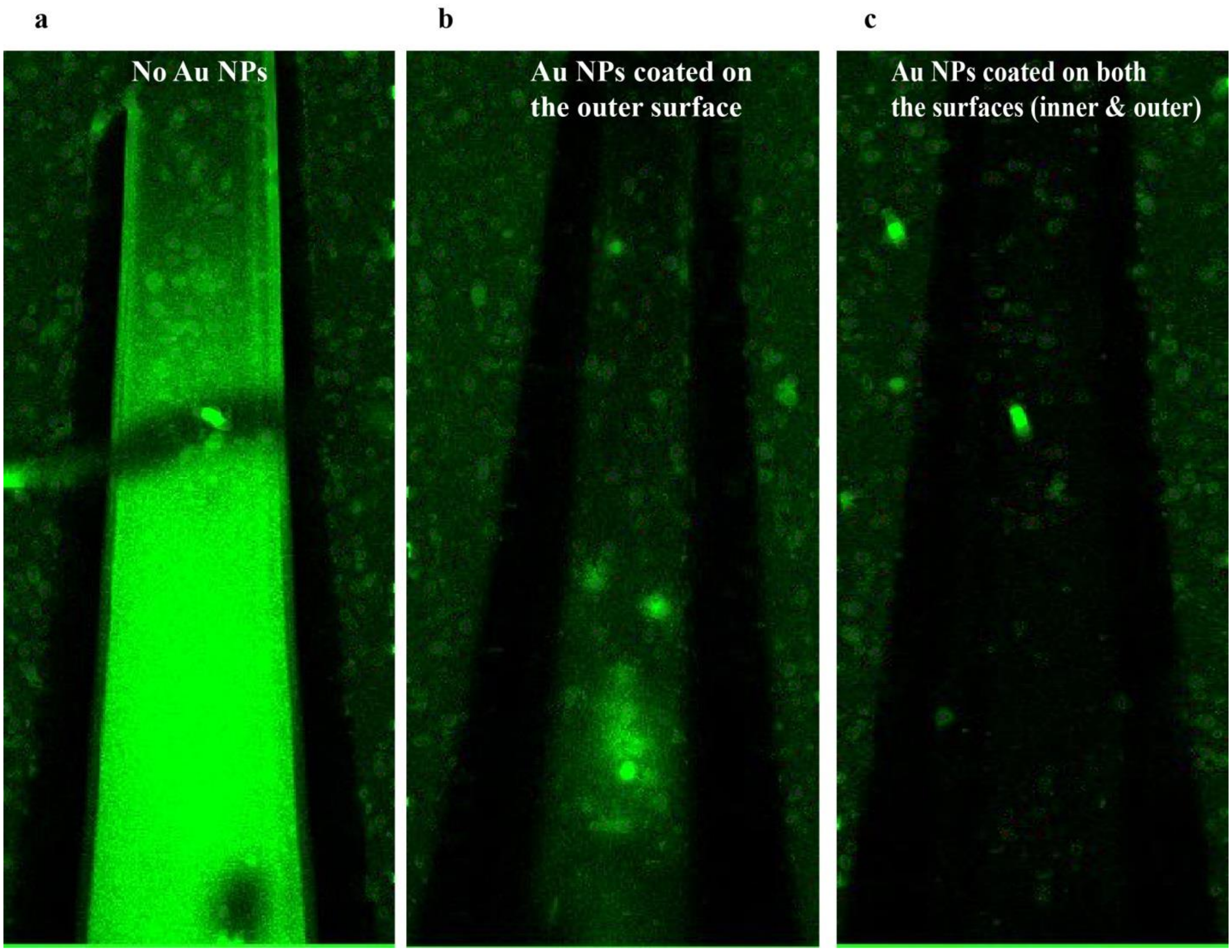

Supplementary Figure S1 | Fluorescence quenching on the surface of the gold nanoelectrodes. Digital micrographs of fluorescence emission of FITC-labeled human serum albumin (FITC-HSA) on glass micropipette tips, viewed emission spectra around 520 nm **a,** uncoated tip **b,** tip coated with Au nanoparticles outside and **c,** tip coated both inside and outside.

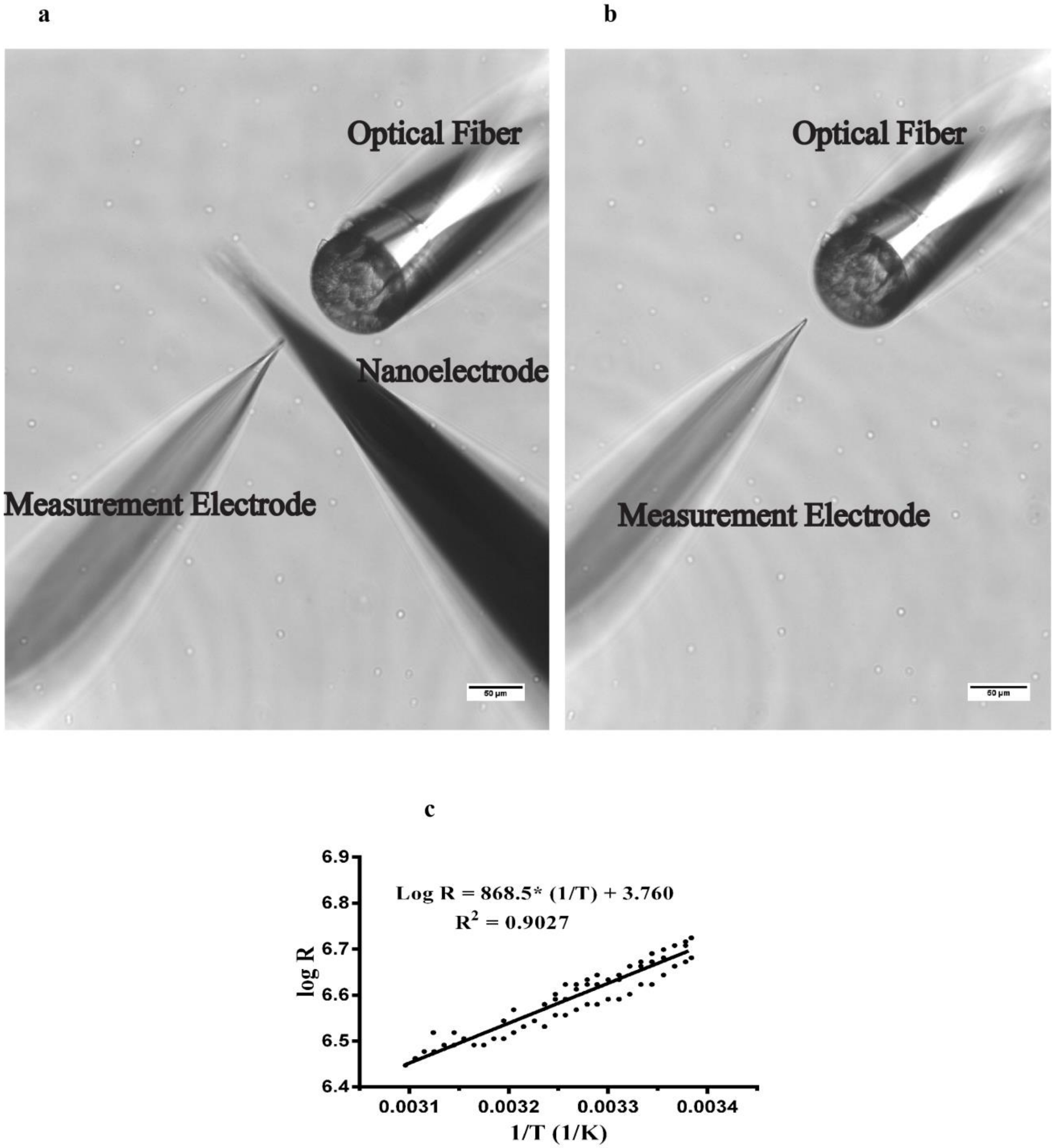

Supplementary Figure S2 | Plasmonic Temperature Measurements. **a,** A digital micrograph taken during the plasmonic temperature measurements at the surface of the nanoelectrode. The micrograph showing the nanoelectrode, measurement electrode, a patch pipette filled with ECS solution and a 50 µm inside diameter optical fiber used to focus the laser beam on the tip of the nanoelectrode. **b,** Digital micrograph of the control experiment, showing the measurement electrode and a 50 µm inside diameter optical fiber. **c,** Micropipette resistance versus temperature calibration curve, showing curve from combined data of three trials. The fitted equation is log R = 868.5 x (1/T) + 3.760 (R^2 = 0.9027).

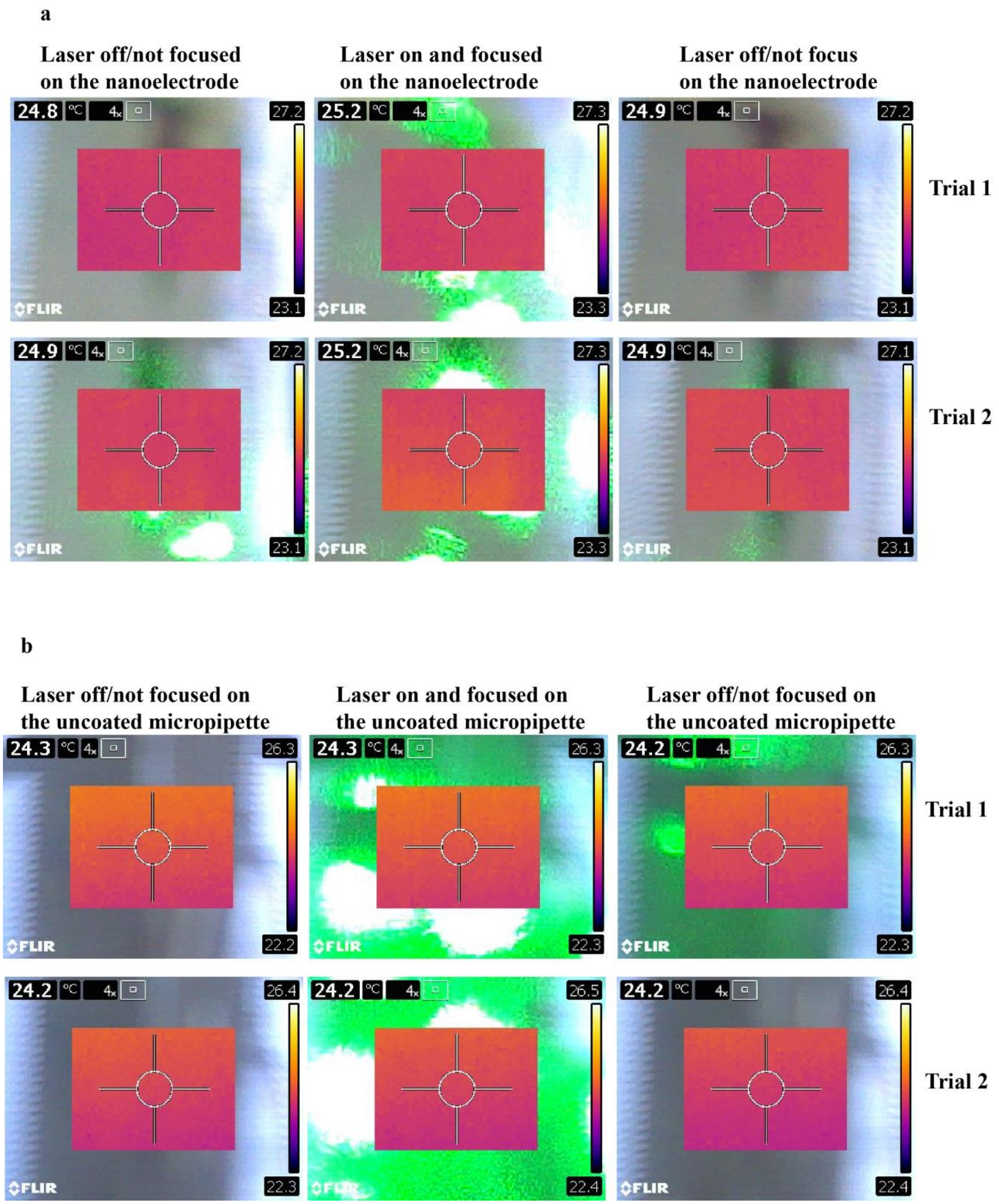

Supplementary Figure S3 | Infrared thermograms of glass micropipettes. a, nanoelectrode – micropipette coated with Au nanoparticles and **b,** control - uncoated micropipettes. Laser was either off or not focused on the tip of the micropipette (left), laser was switched on and focused on the tip of the micropipette tip (middle) and finally, laser was turned off again or not focused on the tip of the micropipette. The shadow in the background shows the micropipette.

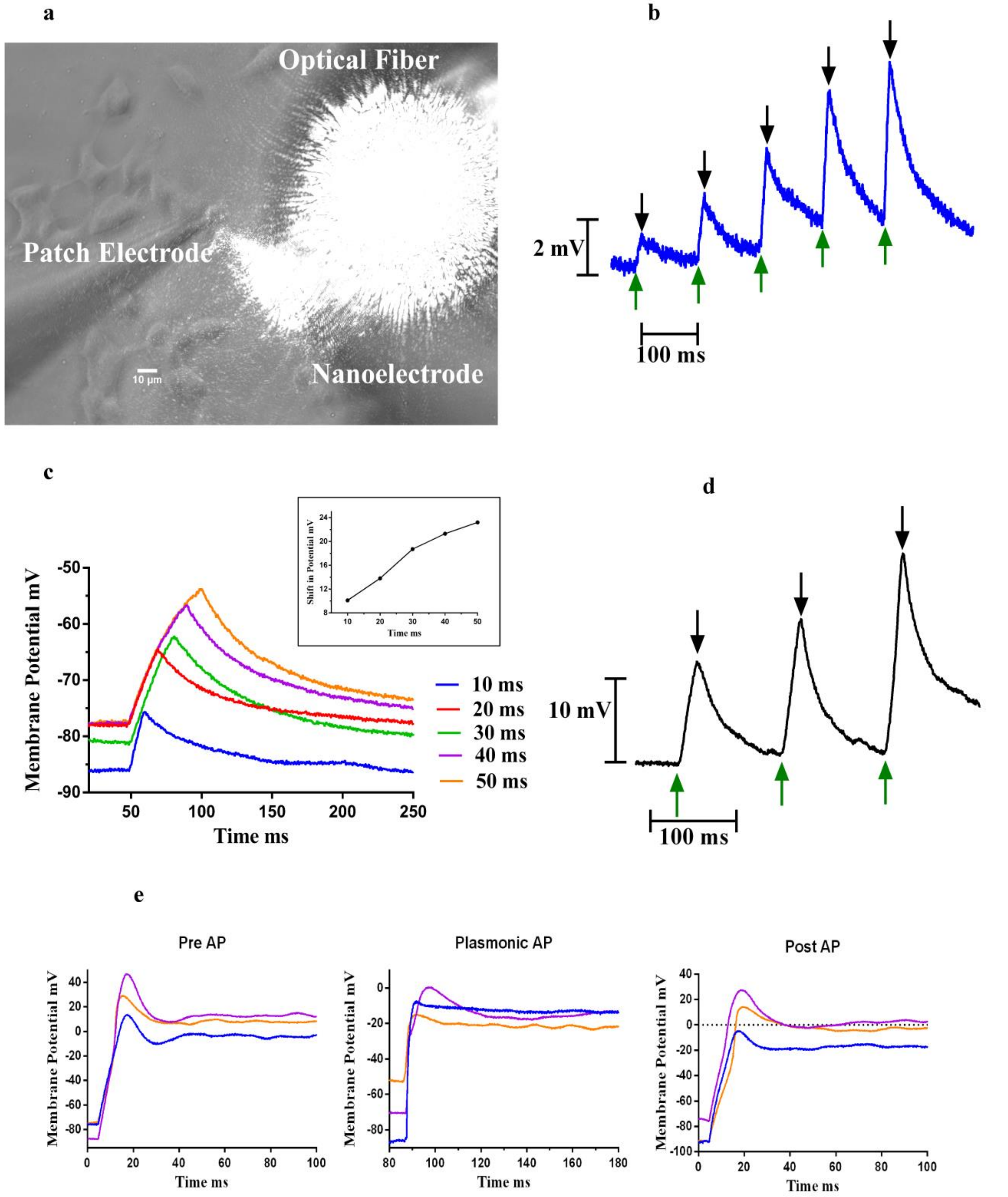

Supplementary Figure S4 | Optical excitatory response – whole cell current clamp recordings. a, A digital micrograph showing the optical stimulation patch-clamp experimental set-up in which the nanoelectrode (glass micropipette coated with Au NPs) was placed adjacent to the cell and a 532 nm green laser was focused on the tip of the nanoelectrode with the help of an optical fiber. Another glass micropipette, patch electrode, filled with ICS was used patch the cell in whole cell configuration and cell response was recorded. **b,** The shift in membrane potential of a representative SH-SYSY cell for 10 ms pulse at five different laser powers; 20 mW, 40 mW, 60 mW, 80 mW and 100 mW (Baseline Potential = -77.8 mV; applied holding current = -23 pA). The shift in potential becomes more prominent as laser power increases. **c,** The shift in membrane potential versus laser pulse timing at 100 mW laser power. **d,** Shift in membrane potential for a representative neonatal cardiomyocyte at three different laser powers; 60 mW, 80 mW, 100 mW for 10 ms laser pulse timing. **e,** Electrically stimulated action potentials (Pre AP & Post AP) were recorded before and after the optical stimulation recordings (Plasmonic AP). The figure shows three different such SH-SY5Y cells.

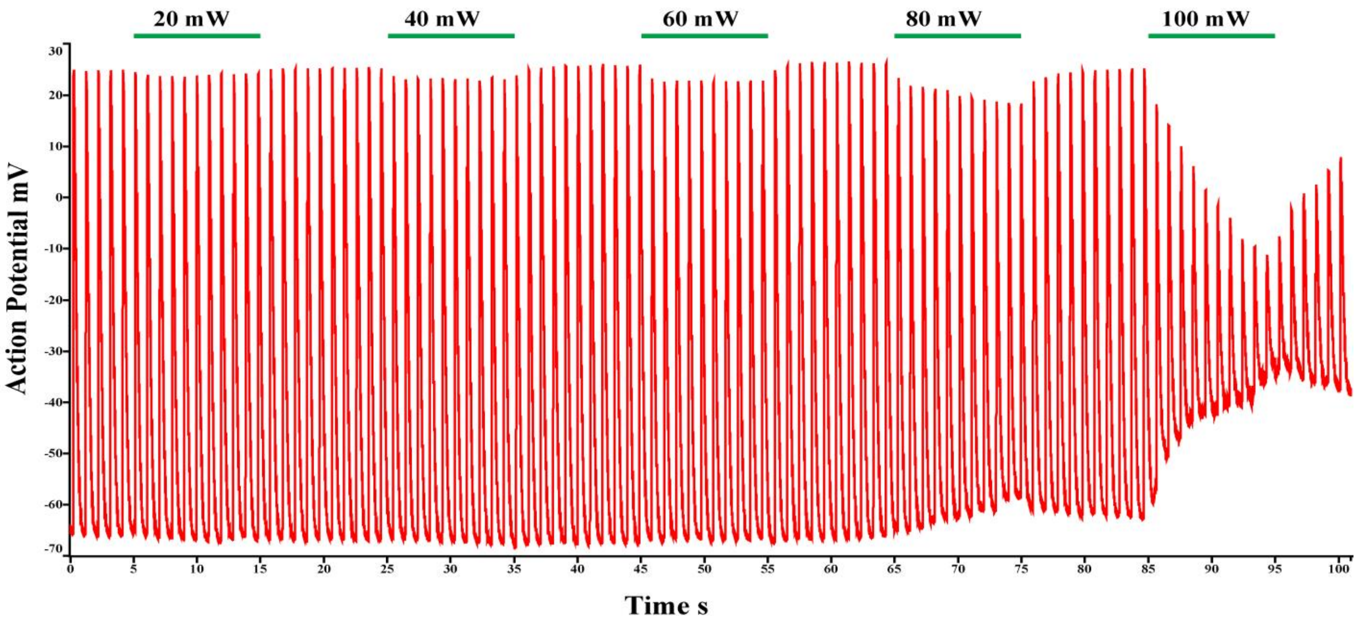

Supplementary Figure S5 | Optical inhibitory response for neonatal cardiomyocytes. Optical stimulation of a spontaneous beating cardiomyocytes. Green bar shows the laser. As power increases, suppression of magnitude of the action potential becomes more significant as shown.

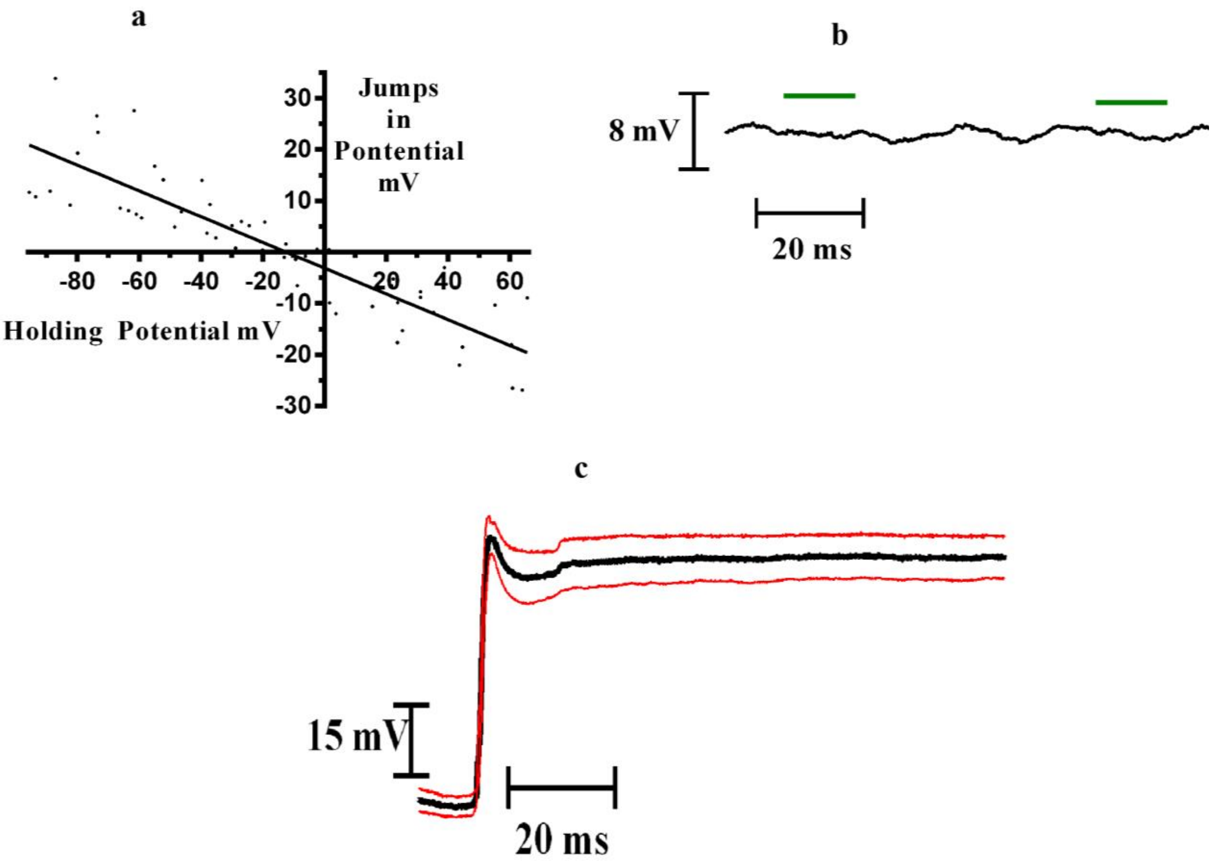

Supplementary Figure S6 | Plasmonic Stimulation for SH-SY5Y Cell – whole cell current clamp recordings. a, The average linear regression curve for holding potential versus jumps when cells were stimulated with 10 ms, 100 mW, laser pulses. **b,** When optical stimulation experiments were done with laser alone, without nanoelectordes, no/little response was observed. Figure shows a SH-SY5Y cells with membrane potential = -53.9 mV. Green bars shows the laser pulse having 100 mW power. **c,** The mean curve of optically stimulated action potentials. Black curve shows the mean values and red curves show the sem values (N=6).

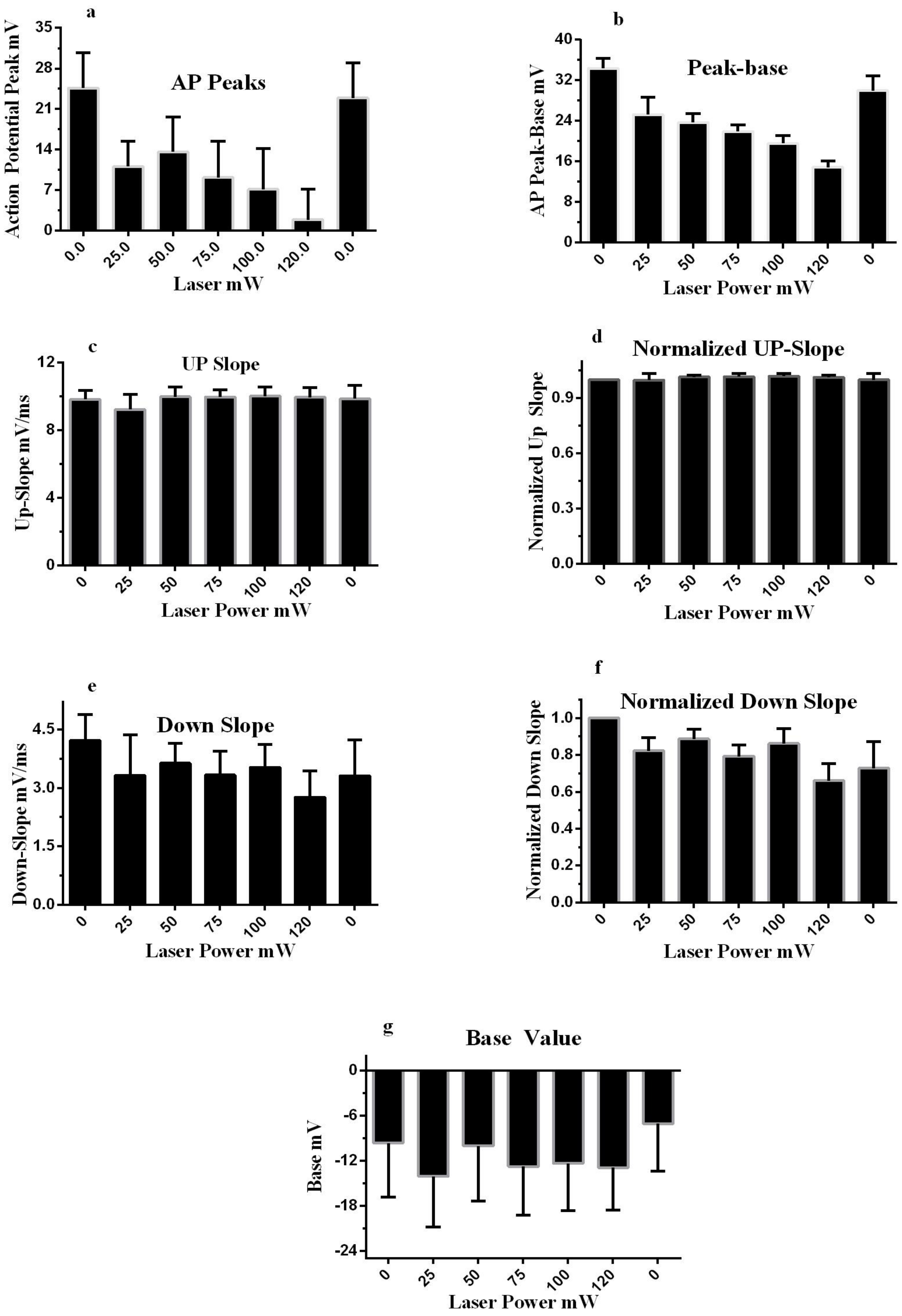

Supplementary Figure S7: Mean data for SH-SY5Y inhibition experiments – whole cell current clamp recordings (One-way ANOVA)). a, b, Action potential peaks and difference between peak value and base value (minima after the peak) decreases with laser power. One way ANOVA was significant for action potential peaks ($F [4, 4] = 153$, $P < 0.0001$) and difference between peak & base values ($F[4, 4] = 40.80$, $P = 0.0007$). Pairwise comparison showed significant difference between the control condition (0 mW) and laser stimulation. . c,d The rate of rise of the action potential did not vary with laser power. Both absolute values and normalized values have the same trends. e, f, Unlike up-slope, the downslope decreases when laser power increases, confirmed by both absolute and normalized values curves. One way ANOVA was significant for downslopes (downslope – $F [4, 4] = 6.014$, $P = 0.0348$; normalized downslope – $F [4,4] = 6.504$, $P = 0.0155$). g, The base value (first minima after peak value of action potential) doesn’t vary. All the data is represented as mean ± sem. The values at zero laser power are electrical stimulations alone.

Supplementary Table 1 | Fitted parameters for plasmonic temperature rise with distance at various laser powers.

**S.N.**	**Laser Power (mW)**	**T_o (K)**	**C (K)**	**k (μm^{-1})**
1	20	4.5	0.7	0.03324
2	40	9.3	2.1	0.03836
3	60	15.0	4.1	0.05593
4	80	20.2	5.6	0.04702
5	100	20.5	6.7	0.03356
